# Pseudocereals in Bakery Products: A Review of Nutritional Composition, Health Benefits and Bakery Applications

**DOI:** 10.3390/foods15081283

**Published:** 2026-04-08

**Authors:** Olivia Atudorei, Denisa Atudorei, Georgiana Gabriela Codină

**Affiliations:** Faculty of Food Engineering, Stefan cel Mare University of Suceava, 720229 Suceava, Romania

**Keywords:** pseudocereals, buckwheat, amaranth, quinoa, chemical composition, health benefits

## Abstract

Pseudocereals are naturally gluten-free crops because they do not contain gluten-forming proteins which are present in other grains. The main pseudocereals used in bakery formulations are buckwheat, amaranth, and quinoa, because they have a balanced nutritional profile including high-quality proteins, dietary fiber, essential minerals, and bioactive compounds with antioxidant, anti-inflammatory, and cardiometabolic health-promoting effects. Due to their high nutritional value, they have increasingly been used as functional ingredients in bakery products, particularly for consumers with celiac disease, gluten intolerance, or those seeking nutritionally enhanced foods. The present paper reviews recent advances on the nutritional, functional, and technological properties of these pseudocereals, focusing on their applications in bakery products. Their influence on dough behavior, product quality, and the nutritional improvement of bread, cakes, biscuits, muffins, and other baked goods is discussed. Also, different aspects of the use of pseudocereals in gluten-free products are presented. Mentions are also made of the fact that the increasing demand for healthier and gluten-free foods highlights the possibility of using pseudocereals as promising ingredients for the development of nutritionally enriched bakery products of acceptable technological and sensory quality.

## 1. Introduction

Pseudocereals have a structure similar to cereal grains and are composed of the aleurone layer and endosperm, which are key structural and nutritional components. The most cultivated pseudocereals in the world are buckwheat (*Fagopyrum esculentum*), quinoa (*Chenopodium quinoa*), and amaranth (*Amaranthus* spp.). These grains have gained increasing global attention due to their nutritional value, agronomic adaptability and versatility in food systems. Amaranth and quinoa are part of the *Amaranthaceae* family, while buckwheat belongs to the *Polygonaceae* family. They are not part of the *Poaceae* family, which includes wheat and barley [1,2,3]. Buckwheat (*Fagopyrum esculentum*), amaranth (*Amaranthus hypochondriacus*), and quinoa (*Chenopodium quinoa*) are important sources of energy due to their high starch content, proteins of high quality rich in lysine and arginine, dietary fiber, and lipids rich in unsaturated fatty acids [4,5].

The use of pseudocereals in bakery products is increasing rapidly. Pseudocereals in milled form can be incorporated into bread, cookies, cakes, crackers, waffles, and muffins, either alone or in combination with other flours, partially replacing wheat flour and enhancing the nutritional value of these products. Refined wheat flour has a lower nutritional value because it retains less of the bran and germ. Flours with a low extraction rate are commonly used in bakery production. Incorporating pseudocereals into flours can significantly enhance their nutritional profile, compensating for the losses associated with low extraction rates [6,7,8,9]. However, it must be taken into account that the additional amount of pseudocereals that can be incorporated depends on their impact on bread and baked products’ characteristics. Due to the fact that these grains do not contain gluten-forming proteins, the rheological properties of the dough during the technological process and the baked products quality are affected.

The potential negative effects depend on the amount of pseudocereal incorporation in bakery products recipes. Therefore, it is necessary to optimize the amount of pseudocereal addition in breadmaking. For example, bread obtained with up to 25–30% pseudocereals incorporated in wheat flour was well received by consumers whereas higher amounts significantly reduced technological and sensory properties of bakery products. For gluten-free products, pseudocereals must totally replace wheat flour or other raw materials which present gluten-forming proteins, their recipe being adapted to obtain good-quality products. They interact with other components, such as hydrocolloids (e.g., guar gum, xanthan gum) and emulsifiers, to improve dough handling, gas retention, and volume in baked goods. This makes it possible to produce gluten-free products with acceptable sensory qualities, including texture, taste, and appearance, while offering superior nutritional benefits compared to conventional gluten-bakery products formulations [10,11,12,13]. In the human body, pseudocereals consumption may improve digestive health due to their high fiber content, which acts as a prebiotic helping in maintaining regular bowel movements, may improve nutrients absorption, and may reduce gastrointestinal risk disorders such as constipation and irritable bowel syndrome [14]. To improve the nutritional profile of pseudocereals, researchers have studied various techniques to increase the bioavailability of nutritional compounds in their composition. Some studies have reported that through germination or fermentation processes, the nutritional compound bioavailability from pseudocereals (mineral elements, vitamins, amino acid, and polyphenols) increased. Also, some anti-nutritional and indigestible factors like phytic acid, lectin, tannin, and protease inhibitors decreased [15]. Both techniques are completely environmentally friendly processes because they do not require the use of polluting techniques.

Traditionally, buckwheat is used as a staple food in many European countries. In Eastern Europe, buckwheat is incorporated into kasha bread, made by combining buckwheat groats or flour with wheat or rye flour, producing a dense, nutty bread, commonly consumed with soups or as a staple food [16,17]. Amaranth has a long history of use in traditional baked and bakery-type foods across various regions of the world. Traditional products such as alegría, made from popped amaranth bound with honey or piloncillo, as well as amaranth-based tortillas, flatbreads, and ceremonial doughs prepared with honey, reflect its cultural and nutritional importance, even though many of these products were not oven-baked in the modern sense. In South Asia, particularly in India and Nepal, amaranth (rajgira or ramdana) was widely consumed during religious fasting periods in the form of flatbreads, fried breads, and biscuits [18,19,20]. Quinoa has been cultivated for thousands of years in the Andean region of South America and was not historically used for leavened bakery products; instead, it was primarily consumed as whole grains, flours, soups, porridges, beverages, and fermented products. Over time, numerous authors have studied the possibility of using quinoa in various bakery products such as bread, cakes and biscuits [13,21,22,23].

This review is relevant because it synthesizes current evidence on the use of pseudocereals in bakery products and their associated nutritional and health-related benefits. For each pseudocereal, this study highlights the chemical composition, associated health effects and applicability in various bakery formulations. This review aims to fill current knowledge gaps by critically integrating evidence on pseudocereals in bakery products. Unlike prior studies that treat nutritional content and technological behavior separately, it examines how compositional traits influence dough functionality and potential health benefits. It also addresses inconsistencies in methodologies and suggests standardized approaches, while highlighting optimal substitution levels and processing strategies to support the development of nutritionally enriched, high-quality bakery products.

## 2. Buckwheat, Quinoa and Amaranth: Raw Materials for Bakery Products

In the process of baking, the main raw material used is wheat flour, of which the main advantage is that it forms gluten during mixing. Although gluten proteins are also present in other cereals, the properties of these proteins do not allow the formation of gluten. This is the main component that affects dough rheological behavior, especially its elasticity and extensibility. The quality of bakery products is influenced by the dough rheological properties, which allow the dough to be molded and to retain its shape when it is molded but also to retain gases [24,25,26,27]. There is a growing interest in the use of alternative natural raw materials in bakery production, driven by consumer demand for products with improved nutritional and functional properties [28].

**Figure 1:**
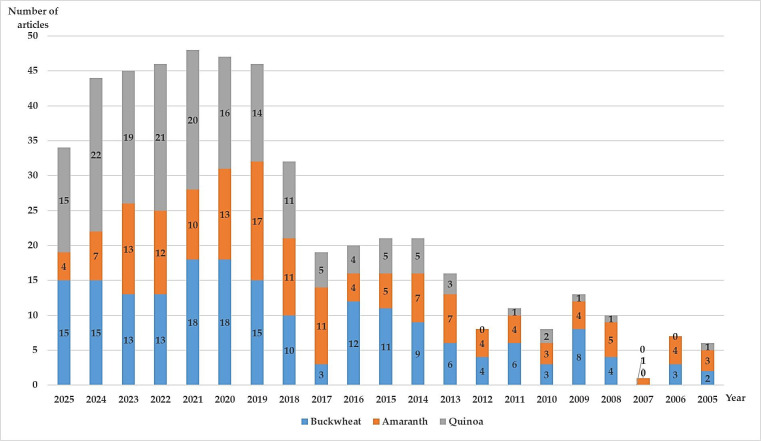
Number of articles published between 2005 and 2025 containing the terms “buckwheat/amaranth/quinoa” and “bakery products”. Source = Web of Science Core Collection (WOS).

Recently, there has been increasing interest in buckwheat, amaranth and quinoa as ingredients in bakery products, as shown in Figure 1. A literature search was conducted in the Web of Science Core Collection (WOS) using the keywords *buckwheat*, *amaranth* and *quinoa* combined with *bakery products*, for articles published between 2005 and 2025. This article reviews their benefits for human health, their effects on the quality of bakery products and their technological performance in the bakery industry.

### 2.1. Botanical and Agronomic Characteristics of Buckwheat, Amaranth, and Quinoa

Buckwheat is most commonly cultivated in Asia, Europe and America. Two species of buckwheat can be distinguished, and they are common buckwheat (*Fagopyrum esculentum* Moench) and tartary buckwheat (*Fagopyrum tataricum* (L.) Gaertn.), these two species being differentiated by their place of cultivation and the amount consumed. Buckwheat is an annual plant, with a deep taproot and a stem branching from the very base, reaching 20–60 cm in height and turning reddish at maturity. The leaves are petiolate, cordate-sagittate, longer than wide. The fruit (nut) is chestnut-brown in color and has three edges. Buckwheat is characterized by considerable genetic variability, which allows it to adapt well to different soil types and climatic conditions. The plant exhibits broad, heart-shaped leaves and produces small, white to pink flowers. Buckwheat is considered a resilient crop, tolerating moderate stress conditions, including poor soils and varying temperatures, while showing resistance to certain pests and environmental disturbances. Buckwheat seeds are classified as pseudocereals because, despite their similarity to true cereal grains in terms of starch content and grain structure, they are botanically dicotyledons. The endosperm constitutes the major portion of the seed [29,30,31,32,33,34].

Amaranth is also a pseudocereal that belongs to the genus *Amaranthus* in the family *Amaranthaceae*. It includes 87 species, of which 17 have been recorded in Europe, 14 in Australia, and 56 in America. It is a plant native to the Americas and has been known for over 7000 years [35]. It is currently cultivated in subtropical and tropical regions. The *Amaranthaceae* family is divided into two sections, *Amaranthus saucer* and *Blitopsis dumort*, with almost the same number of species. Species are categorized according to their primary use as a vegetable, as a cereal, as an ornamental plant or as a weed [36,37]. Amaranth, together with quinoa, is a crop characterized by high genetic variability, which contributes to its excellent adaptability to different soil types and climatic conditions. The color of amaranth varies from light to dark green, with an elongated to elliptical shape. Amaranth is a dicotyledonous species [37], and it is considered a convenient cereal due to its unpretentious conditions, resisting high temperatures and drought very well. This pseudocereal is also highly resistant to pests or other disturbances that may occur. Amaranth seeds are considered pseudocereals because of their similarities to real cereal grains, monocotyledons, as well as their important starch content, which is located in the perisperm, while the germ constitutes most of the seed’s structure; for this reason, amaranth seeds are considered dicotyledons and therefore pseudocereals [38,39,40].

Quinoa is found under the scientific name of *Chenopodium quinoa Willd*. This is a pseudocereal that is advantageous from an agricultural point of view because it has good adaptability to different altitudes and types of climates. Thus, it is cultivated in areas with high altitudes but also in those that are prone to frost [41], being tolerant to saline soils and drought conditions. Quinoa is naturally gluten-free, as it does not contain the gluten-forming proteins present in wheat and, for this reason, is suitable for individuals with gluten intolerance [42]. Quinoa grains are usually round, small and flat and have a white color, but there are also species that are red or black. It is currently cultivated in South America but also in the United States, China, Europe, Canada and India, from sea level to altitudes higher than 3800 m. In Europe, it was first brought to England in 1970 [43,44,45]. Quinoa has a sweet, nutty taste, and when it is cooked, it becomes softer. Quinoa grains have a spherical shape with a diameter of 1.4–1.6 mm [46,47].

### 2.2. Nutritional Composition of Pseudocereals

Buckwheat grains are a good source of nutritional compounds, and for this reason they are used as an ingredient in food stuff such as bakery products, pasta, biscuits, pastries and confectionery. Unlike most cereals, buckwheat contains 60 ÷ 70% starch, 10 ÷ 12.5% protein [48,49] and many antioxidants, minerals (K, P, Zn, Mg), dietary fiber [16] and bioactive compounds including flavonoids like quercetin and rutin, which exhibit strong anti-inflammatory, antioxidant, and vascular-protective properties [50]. Buckwheat also contains significant quantities of B vitamins, particularly niacin and riboflavin. Adding buckwheat to bakery products leads to the improvement of their nutritional quality, also due to the presence of essential amino acids necessary for the human body. Buckwheat is high in lysine, threonine, and sulfur amino acids of which wheat flour presents deficiency. For this reason, the incorporation of buckwheat into bakery products is beneficial [51,52,53]. Buckwheat seeds contain a relatively low amount of lipids which range between 2 and 3%, with minor variations depending on variety and growing conditions. The lipid profile has a high amount of unsaturated fatty acids such as oleic acid (ω-9), linoleic acid (ω-6), and α-linolenic acid (ω-3) [54,55]. Buckwheat lipids also contain minor important constituents such as tocopherols, phytosterols, and phospholipids. Some bakery products do not fully meet the body’s nutritional needs, and buckwheat’s high content of essential nutrients and bioactive compounds makes it an effective solution for preventing these deficiencies and supporting the proper functioning of the body [56,57].

Amaranth is a pseudocereal with a very balanced nutritional profile. Studies in the field have highlighted the fact that amaranth has a high content of essential micronutrients, namely vitamin C, iron, calcium, folic acid, and β-carotene. The most important chemical compounds in amaranth seeds are proteins and minerals. The protein content, depending on the species, varies between 11 and 20% [58,59,60,61], thus being higher than the protein content in rice (~7.5%) [62], corn (~10%) [63] and wheat (~14%) [64]. The most important proteins in amaranth seeds are globulins and albumins, and they represent 60–70% of the total protein nitrogen [65]. Amaranth contains twice the lysine content of wheat proteins [4]. The dietary fiber content of amaranth seeds varies between 9 and 16.5% depending on the species, this content being higher than that of corn, wheat, oats, rice, and sorghum [66]. Insoluble dietary fiber in amaranth seeds represents 75–88% of total fiber, which makes them recommended for high-fiber diets. The high value of mineral content is attributed to the presence of phosphorus, potassium, and calcium [65,66,67,68]. An interesting feature of the amaranth plant is that the leaves are also edible, with a high content of calcium (267 mg/100 g) and vitamin C (80 mg/100 g), the latter having almost three times more calcium than spinach. The lipid content of amaranth seeds ranges from 5 to 8% and is well-known as a natural resource particularly rich in squalene, which accounts for 2.4–11.0% of the total lipid content, depending on the species [69,70]. Squalene is an unsaponifiable fat that acts as a precursor for the biosynthesis of all steroids in animals and plants. Among the health benefits attributed to squalene are the reduction of cholesterol levels and the reduced risk of developing various types of cancer [71]. The main fatty acids present in amaranth oil are linolenic and oleic acids (omega 3) [72]. Amaranth seeds also contain tocotrienols. Like cereals, amaranth contains starch, a major component, with a content ranging from 48 to 69 g/100 g, depending on the species [60]. Research has shown that amaranth starch has high solubility, low viscosity and gelatinization temperature [61,73]. Amaranth seeds are also an excellent source of niacin (4.6 mg/100 g), vitamin E (5.7 mg/100 g) and riboflavin (0.70 mg/100 g seeds) [69,70,71,72,73,74,75].

Quinoa, from a nutritional point of view, is distinguished by its high protein content and important amino acids. The nutritional value of quinoa grains varies depending on the grain variety and the origins of the soil in which it was grown. Studies in the field have shown that the nutritional profile of quinoa grains is distinguished by a balanced profile in amino acids such as lysine, threonine and methionine. At the same time, it contains important values of lipids (1.8–9.5%), dietary fiber (7–14%), phenolic compounds, vitamins, and minerals [46]. The protein content of quinoa grains varied from 12% to 22%, a percentage that far exceeds the amount of protein contained in other cereals but is lower than in legume seeds. The World Health Organization (WHO) has shown that the proteins found in quinoa have characteristics like those in milk. In addition, the amount of amino acids contained is of a higher quality than that of wheat, barley, rice or soybeans. Unlike other cereals or legumes, quinoa is a complete source of protein, containing eight essential amino acids: leucine, isoleucine, lysine, phenylalanine, methionine, tryptophan, threonine and valine [76,77,78,79]. The carbohydrates contained in quinoa are monoglucosides and diglucosides, oligosaccharides, and polyglucosides. Starch, the main representative of the carbohydrates contained in quinoa, is present in the grain in a proportion of 32–69.2%. Regarding the lipid content, compared to grasses, quinoa has higher amounts of monounsaturated and polyunsaturated lipids. Triglycerides are present in the highest amount, representing approximately 50% of the total neutral lipids, and diglycerides represent 20%. Linoleic acid is a polyunsaturated fatty acid present in the highest amount in quinoa with positive effects on cardiovascular diseases [45,80]. Quinoa also contains valuable micronutrients, such as vitamins and minerals. The most important minerals in this pseudocereal are potassium, phosphorus and magnesium, to which calcium, iron, copper, zinc and sodium are added. It also contains vitamins from the B group (B1, B2, B3, B6), vitamin E and numerous antioxidants. Quinoa has in its composition essential constituents necessary for baby food and can be easily consumed by children on a diet [48].

Therefore, it can be concluded that the nutritional composition of pseudocereal grains, compared to conventional cereals, is superior, making them suitable for use as an addition to the recipe for manufacturing bakery products in order to optimize their nutritional value. The three pseudocereals studied exhibit a superior nutritional profile compared to wheat, which is the most widely used cereal in the baking industry and are characterized by higher protein quality due to their increased lysine content. Furthermore, considering the values presented in Table 1, pseudocereals also show higher levels of dietary fiber and essential minerals such as calcium, magnesium, and iron, representing a valuable nutritional alternative to wheat. The chemical composition of the buckwheat, amaranth and quinoa is shown in Table 1.

## 3. Health Benefits of the Pseudocereals on the Human Body

Since ancient times, plants have been used to treat certain diseases and conditions. Currently, there is increasing emphasis on the healing brought about by the consumption of certain foods or by supplementing them with certain additions that improve their nutritional profile. It is intended that these foods also bring an increase in the content of certain compounds that have a positive influence and manage to improve or even treat certain diseases. There is an increasing desire to support and complement conventional medicine by consuming foods that bring benefits to human health. The number of people suffering from celiac disease is increasing; therefore, special attention is paid to the consumption of gluten-free foods and the obtaining of products that satisfy all preferences.

### 3.1. Buckwheat

Buckwheat is a pseudocereal with multiple health benefits that has been studied in the scientific literature. Buckwheat has considerable nutraceutical and nutritional properties. It has been shown to exert physiological effects, including blood glucose regulation, cardiovascular protection, antioxidant activity, modulation of lipid metabolism and the intestinal microbiota, and it also has applications in functional nutrition.

Blood pressure is regulated through the consumption of buckwheat. This is possible due to buckwheat’s high magnesium content, which helps support vascular relaxation, thereby improving circulation and distribution of nutrients as well as lowering blood pressure, resulting in the optimum combination for a healthy cardiovascular system [48]. Additionally, flavonoids such as rutin and other polyphenols contribute to vascular protection and reduce capillary fragility [95,96]. The nutrients in buckwheat contribute to maintaining blood sugar. In a study comparing the effects of bread made from buckwheat flour versus those obtained from wheat flour on blood sugar, bread containing buckwheat flour was found to reduce both blood glucose and insulin levels [97]. A single dose of buckwheat seeds can lower blood glucose levels by 15 ÷ 19% after 90 ÷ 120 min from the time of consumption [98]. Blood pressure is also reduced due to the high content of essential flavonoids (vitexin, isovitexin, isoorientin) and anthocyanins, such as cyanidin-3-O-rutinoside and cyanidin-3-O-glucoside [99,100]. The reduction in blood pressure was studied through the consumption of food products based on a mixture of buckwheat flour and rice flour, which showed positive effects and also reduced the risk of type 1 diabetes [101]. The antihypertensive effect was also studied through tests conducted on rats that were administered via a pasta recipe made from buckwheat sprouts (30%) and durum wheat semolina (70%) [102].

Studies have highlighted that the consumption of buckwheat also has positive effects on cholesterol levels. Tests on hamsters concluded that buckwheat flour reduced the level of cholesterol non-HDL, the level of total cholesterol in plasma and the level of cholesterol in the liver. The effect of buckwheat consumption on lowering cholesterol was studied using bread supplemented with 50% buckwheat flour. The study demonstrated that the final product was rich in quercetin, quercetin-glucoside, protocatechuic acid, and rutin—compounds that prevent increased capillary fragility associated with hemorrhagic diseases and reduce blood pressure and cholesterol [96,103].

The consumption of buckwheat is not limited to these benefits; it has been shown that, due to its composition, buckwheat is included in various menus, in diets for numerous health conditions, and recently, it has also been introduced into weight loss diets. It has been found that a diet rich in buckwheat stimulates the multiplication and activity of bifidobacteria and lactobacilli in the colon, making buckwheat a successful prebiotic. Buckwheat antioxidant substances have a preventive role in the occurrence of cardiovascular diseases, cancer, and neurodegenerative diseases associated with oxidative stress. Compared to biscuits made from wheat flour, biscuits made with buckwheat flour had a higher content of antioxidant activity and total polyphenols, without affecting sensory characteristics, which supports the use of buckwheat flour in gluten-free products with functional food benefits [98,104,105]. Due to its remarkable nutraceutical properties and content of antioxidant compounds [95], buckwheat is considered by specialists as the “golden crop” of the future [106]. The multiple health benefits, such as alleviating inflammatory bowel diseases, reducing the risk of type 2 diabetes and chronic intestinal disorders, and also improving nutritional value in terms of mineral content, antioxidant activity, and alpha-linolenic acid levels, have been highlighted through the consumption of bread made from buckwheat flour (40%) and enriched with wheat flour, rye flour, and chia flour [100].

Although buckwheat contains significant amounts of rutin and flavonoids, these compounds are sensitive to high temperatures. Thus, baking can significantly reduce their concentration. Their bioavailability depends on the food matrix, and typical consumption may be insufficient to reproduce the clinical effects observed in studies. For this reason, further research is needed to determine effective doses in a regular diet.

### 3.2. Amaranth

Amaranth is a pseudocereal with significant health-promoting properties and is widely applied in gluten-free dietary products. Studies have shown that amaranth provides important health benefits, such as regulating blood pressure, improving glycemic control, anti-inflammatory effects, reducing cholesterol, supporting digestive health, and preventing coronary heart disease. The formation of malignant cells is prevented by the amino acid lysine, iron, vitamin E, magnesium, potassium, phosphorus, and vitamin C, which help reduce free radicals responsible for the development of these cells. Additionally, it improves the functioning of the immune system [107,108].

Also, researchers have studied the effect of amaranth on cardiovascular health. In this context, gluten-free cookies containing amaranth were consumed. The study found that these cookies can help prevent the formation of blood clots due to the bioactive peptides released during gastrointestinal digestion. At the same time, the peptides play a role in inhibiting the angiotensin-converting enzyme, which is responsible for regulating blood pressure. Thus, consuming gluten-free cookies may contribute to lowering blood pressure. The study also highlights the antithrombotic activity of these cookies, thereby reducing the risk of a heart attack or stroke [109]. Due to its squalene content, it may be used in the prevention of coronary heart disease [44,55,60,68]. However, to confirm the physiological relevance of these experimental findings, clinical studies in human subjects are required.

In addition, amaranth has been found to possess anti-allergic and antioxidant properties. However, amaranth grains have a high glycemic index due to their easily digestible starch. To maximize its benefits, it is recommended to use amaranth flour in a mixture with nuts, seeds, flours from other cereals, and dried vegetables. Studies in the field report that amaranth has also been used for the treatment of cardiovascular diseases, diabetes, renal failure, gynecological disorders, jaundice, and other infections. Tests conducted on rats concluded that wholemeal bread with amaranth can reduce the risk of cardiovascular disease, improve the lipid profile, and contribute to lowering blood pressure. Additionally, bread consumption has beneficial effects on the glycemic index and helps regulate body weight, likely due to its high content of insoluble dietary fiber [110,111].

Even though amaranth contains significant amounts of polyphenols and saponins, these compounds can be partially degraded during baking or other thermal processes. The bioavailability of polyphenols and saponins may be reduced in processed products, and the doses used in intervention studies are often higher than typical consumption. It is therefore very important to conduct long-term clinical studies to confirm their physiological efficacy.

### 3.3. Quinoa

Along with the other two pseudocereals, quinoa also provides health benefits, notably beneficial effects on chronic diseases such as obesity, diabetes, celiac disease, and anemia [107,112]. Over time, numerous authors have studied the possibility of using quinoa as an ingredient in various food products. The goal was different, depending on the intended purpose: obtaining new food products, improving the nutritional profile of some food products, obtaining foods for people with celiac disease.

Quinoa has significant potential to improve consumer health. For example, high amounts of phytosterols in quinoa seeds can lower cholesterol levels, presenting anti-carcinogenic, antioxidative and anti-inflammatory properties. The high antioxidant capacity of quinoa is also due to its high phenolic compounds, which vary between 0.46 and 1.84 mg/g of dry weight [84]. The addition of 25% quinoa flour to bread enriched its polyphenol content, which led to increased antioxidant activity and contributed to the body’s protection against oxidative stress, thereby having positive effects on health [113,114]. Quinoa presents a high starch amount characterized by high digestibility and solubility. The non-starchy polysaccharides are fibers which are higher than in other cereals, with more than 80% of them being insoluble. However, compared to other cereals, quinoa presents more soluble fiber primarily composed of galacturonic acid, glucose, and arabinose. Quinoa’s high fiber content may lower the risk of constipation and colon cancer [112,115].

**Figure 2:**
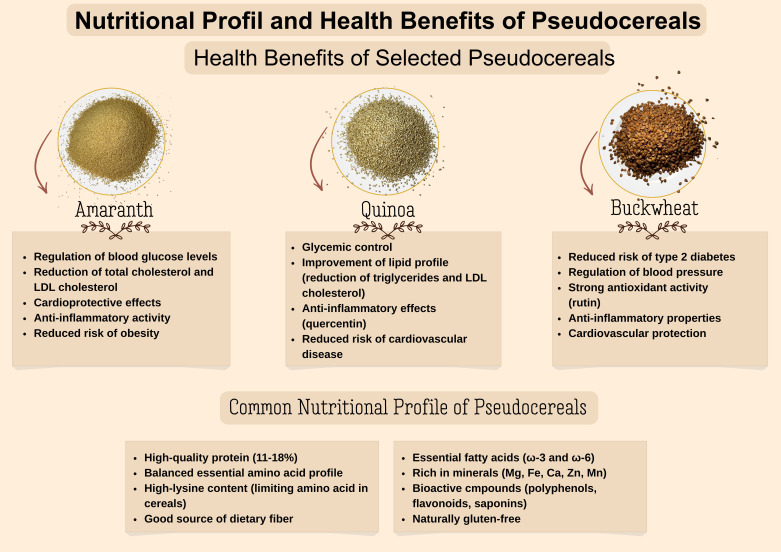
Nutritional profile of pseudocereals and their impact on human health.

Quinoa is a good source of essential fatty acids like linolenic and linoleic, which are necessary for the human body but cannot be synthesized endogenously, and presents a range of important health benefits. Quinoa is also a high source of vitamin A, riboflavin, folic acid, vitamin B6 and vitamin E which support skin health, heart protection, improvement of the immune system, reproductive health and fetal development, and a reduction in oxidative stress [107,108]. Researchers in the field have studied the effects of quinoa on lipid profiles and glucose control. By replacing the cereal with quinoa, this leads to an improvement in the controlled attenuation parameter score, low-density lipoprotein cholesterol levels, and insulin resistance (HOMA-IR), while also leading to reductions in body weight and waist circumference. Regarding liver enzymes and high-density lipoprotein cholesterol, no significant changes were observed. The consumption of quinoa cookies (15 g/day, containing 60% quinoa flour) led to an improvement in the total cholesterol/HDL ratio, reduced total and LDL cholesterol, as well as a decrease in body weight and BMI. Therefore, the consumption of quinoa cookies may contribute to reducing the risk of cardiovascular diseases in older adults [116,117]. Additionally, bread enriched with quinoa (20 g of flour/day) led to a significant decrease in postprandial glycemic response, demonstrating positive effects on glucose control, with minimal effects on other cardiovascular risk markers [98]. An overview of pseudocereals’ nutritional composition and their impact on health benefits is shown in Figure 2.

Although quinoa is a pseudocereal rich in bioactive compounds and exhibits multiple health benefits, thermal processing, such as baking, can reduce their concentration. The bioavailability of nutrients depends on the food matrix and doses used in experimental studies. This may be higher than typical consumption. Long-term clinical studies are necessary to fully assess how processing affects their physiological efficacy.

Alvarez-Jubete et al. (2010) [118] comparatively analyzed wheat bread samples and gluten-free samples obtained using pseudocereals. The study concluded that, although thermal processing reduces the total phenolic content, the pseudocereals have a positive effect on the nutritional composition of the final product compared to products without pseudocereal addition. For example, in the case of buckwheat-based samples, after baking, the total phenolic content decreased from 323 to 64.5 mg GAE/100 g d.w., while in wheat bread samples a reduction from 53.1 to 29.1 mg GAE/100 g d.w. was observed. Therefore, although products enriched with pseudocereals provide a considerable dietary intake of polyphenols, further studies are necessary to determine whether typical serving sizes truly supply sufficient amounts to confer significant health benefits [118].

Gil et al. (2021) [114] studied the effect of baking on polyphenol content and antioxidant activity. The researchers aimed to determine whether the addition of quinoa could improve the functional profile of the bread compared to that made from refined wheat. The authors concluded that bread containing 25% quinoa flour of a black variety has a total polyphenol content 12.8 times higher than that made with refined wheat and also exhibits approximately three times greater antioxidant activity. Thus, after baking, the content of extractable phenolic acids largely decreased in both types of samples, indicating sensitivity to heat treatment. In contrast, flavonoids are more stable and, in many cases, increase significantly after baking due to the release of bound (hydrolyzable) forms. Moreover, the study highlights that rutin was not detected in bread made from refined wheat, whereas in quinoa bread, it was found that quercetin content is approximately 64 times higher than in wheat bread. Regarding bioavailability in the food matrix, the baking process can reduce the content of some heat-sensitive compounds, but it may increase the extractable fraction of flavonoids, suggesting a potential improvement in their availability for absorption. Although the effect of baking on polyphenol content and antioxidant activity cannot be inferred from concrete clinical results, the considerable quantitative difference between bread made with quinoa and that made with refined wheat indicates that incorporating this pseudocereal into various food products may meaningfully contribute to the daily intake of polyphenols [114].

## 4. Techno-Functional Properties of Pseudocereals in Bakery Products

Pseudocereals have distinct techno-functional properties, which can have an important impact on the dough and finished bakery products. First of all, it is known that pseudocereals do not contain gluten-forming proteins, which has a major impact on the dough matrix but also on the quality of bakery products. Wheat grains contain the storage proteins gliadin and glutenin, which interact and form gluten following hydration during the kneading process. Gluten is a viscoelastic protein network that determines the technological properties of dough and the final quality of bakery products. Gliadin is responsible for giving the elasticity and extensibility specific to dough, and glutenin provides it with cohesion and strength. Thus, the interaction between the two determines the specific technological properties of dough and has a direct influence on its technological properties. The gluten network is an elastic and extensible three-dimensional structure, capable of retaining gases produced during fermentation, which gives baked goods their specific volume and crumb porosity [119,120].

Studies in the field have shown that a maximum of 45% of the replacement of wheat flour with gluten-free flour would be acceptable in order not to negatively modify dough handling ability in terms of stickiness, dough hardness, cohesiveness, adhesiveness and springiness [121]. In the absence of gluten, starch becomes the main structural component. Starch in wheat flour plays an important role in the gelatinization process that occurs during baking. It mainly absorbs water and interacts with the gluten network, stabilizing the gas in the system. It also has a direct influence on the elastic and aerated texture of bread. Gluten is what forms the elastic structure around the gas bubbles, and starch absorbs water and gels during the baking process, which stabilizes the structure created by gluten. If gluten is absent from the matrix, even if starch gelatinizes, gas bubbles cannot be effectively retained, resulting in a lower specific volume and reduced porosity of the bread. The starch in pseudocereals has a different profile because the ratio between amylose and amylopectin is different [122], which influences the gelatinization process and implicitly the elasticity of the crumb. For example, in the case of buckwheat, the major component is starch, characterized by low gelatinization temperature, high water absorption capacity, and moderate viscosity, making it suitable for different bakery product processing applications, including gluten-free products [123]. Rheological studies indicate that buckwheat starch exhibits pseudoplastic and shear-thinning behavior, which contributes to improved dough handling and texture in baked goods compared to some conventional cereal starches [122,123,124]. Buckwheat proteins exhibit good water-binding and emulsion-forming capacities, as well as thermal aggregation and gelation behavior when modern techniques such as alkaline treatment, heating, or ultrasonication are applied, which facilitate covalent interactions. Covalent protein complexes can be formed through the irreversible binding of proteins with various compounds, such as polyphenols and carbohydrates [125]. These characteristics make buckwheat flour a beneficial ingredient for many food products. Although most of the biochemistry and chemistry of buckwheat flour (and the related nutritional and health benefits) have been well investigated and understood, much of the mechanism underlying the technological properties and potential uses of this pseudocereal is still under investigation [48].

Amaranth has not only been studied for its nutritional compounds but also to determine their properties. An important example in this context is the use of amaranth as an emulsifier, which is the basis of many applications in the food industry. The rheological properties of amaranth starch indicated that it possesses pseudoplastic and thixotropic behaviors, and the flow properties were better than those of corn starch [126]. In a study by Woo et al. (2025), in which the effect of sourdough made from amaranth protein isolate on the quality of gluten-free bread made from buckwheat flour was tested, it was concluded that the addition of amaranth protein isolate favored the formation of the protein network, which led to a positive impact on the rheological and structural properties of the dough, thus improving the quality of the gluten-free bread [127]. Quinoa flour has a higher water-binding capacity than wheat flour. This is mainly due to the high content of dietary fiber, the finer granular structure of the starch, and also to the hydrophilic gluten-free proteins. This will lead to competition for water absorption between the components of the quinoa grains and the gluten proteins of wheat. As direct consequences, the formation of the gluten network will be delayed; the dough will have an increased viscosity and a reduced extensibility. Also, the bread will have a lower specific volume and the crumb will have a denser structure and a more pronounced gumminess [128,129]. In pseudocereal bread, the absence of gluten prevents the formation of an elastic protein network capable of retaining gas in the system and binding water in the dough. In this case, starch becomes the main component, and the texture of the bread depends largely on the gelatinization process during the baking stage. Starch has the capacity to absorb significant amounts of water, but water binding is unstable in the absence of gluten, which will lead to its migration and a rapid hardening of the bread crumb. The significant amount of lipids can have a positive effect on the retrogradation of starch due to the formation of complexes, but these lipids cannot fully compensate for the substitution of gluten [130].

In an attempt to develop gluten-free products with high-quality characteristics, Poshadri et al. (2023) [131] concluded that a mixture consisting of 50% amaranth flour, 40% buckwheat flour, and 10% quinoa presents the best alternative. The study was conducted in comparison with wheat flour, and the following parameters were determined: water absorption capacity, oil absorption capacity, swelling power, foaming capacity, bulk density, water holding capacity, and water solubility index. The authors of the study concluded that the water holding capacity, water solubility index, bulk density, swelling power, and oil absorption decreased with increasing buckwheat and decreasing quinoa amount. In contrast, the value of the water absorption capacity parameter increased with the increase in the amount of buckwheat flour in the composite flour formulations [131].

Given the considerations above, it is recommended to carefully control the percentage of pseudocereal flour in bread formulations to preserve dough rheology and bread quality, while maximizing the benefits of pseudocereal addition.

## 5. Bakery Applications

### 5.1. Buckwheat

Buckwheat flour is often used as a partial substitute for white wheat flour due to its technological and nutritional advantages. In bread recipes, buckwheat flour incorporations in percentages of 10–30% are increasingly encountered. Studies have shown that percentages of up to 50% make it possible to obtain dough with good rheological characteristics and bread samples with improved specific volume, and even a percentage of the addition of 85% offers the opportunity to obtain products with acceptable sensory characteristics including color, texture and odor [132,133]. In studies conducted so far, the incorporation coincided with a reduction in hardness, gumminess and chewiness of bread and an improvement in the crumb characteristics and in the specific loaf volume [51]. Bread containing approximately 15% buckwheat flour also showed increased levels of bioactive compounds such as rutin and quercetin together with enhanced antioxidant activity [51,132]. Buckwheat flour particle size also has an influence on bread quality. Studies have reported that particle sizes larger than 180 μm were reported to produce bread with improved specific volume and crumb porosity [134]. Buckwheat flour can also be used in sprouted form, with a percentage of 10–30% resulting in lower hardness and higher loaf volume, compared with bread with unsprouted buckwheat. However, it has been shown that extending the germination time of buckwheat grains and using high percentages can reduce gluten extensibility, and therefore a percentage of 10% for bread and 30% for crackers is recommended as appropriate [135]. Cakes containing up to 30% buckwheat flour were also reported to be well accepted by consumers [136,137].

Because buckwheat does not contain gluten-forming proteins, it is often used to develop gluten-free bakery products that are suitable for people with gluten intolerance, celiac disease or gluten allergy [138,139,140,141]. There are several examples of this, such as bread produced using a premix consisting of buckwheat flour, chia flour and xanthan gum, which allows the bread to maintain very low gliadin levels (<1 mg/kg) [142]. Similarly, gluten-free bread produced from tartary buckwheat flour and chia flour showed significantly improved nutritional value, including higher levels of α-linolenic acid, dietary fiber, protein, ash and antioxidant capacity together with lower carbohydrate content [142,143]. Increasing the proportion of buckwheat flour (15–45%) in mixtures with rice and cassava flours improved bread quality, including higher insoluble fiber content, increased porosity and greater specific volume, while maintaining good sensory acceptability [144]. In addition, the incorporation of 10% teff flour combined with rice or buckwheat sourdough was reported to further enhance bread acceptability [145].

In addition to bread, buckwheat flour is also used in other bakery products, for example, gluten-free biscuits showed higher oil absorption capacity and improved sensory characteristics when hydrocolloids such as acacia gum, guar gum, tragacanth gum or xanthan gum were used [146]. Samples with acceptable quality were obtained when 20% buckwheat flour was incorporated into gluten-free cookies based on rice flour [147]. The germination process was shown to have a positive effect in the case of biscuits obtained from buckwheat and rice flour; this was due to reduced starch retrogradation [148]. López et al. investigated spontaneous sourdough prepared from buckwheat and quinoa flours at addition levels of 10–20% and concluded that the incorporation of 20% sourdough provided the best technological and sensory performance [149].

### 5.2. Amaranth

On the one hand, a low percentage of amaranth flour addition, in percentages of 5–15%, has been reported in the literature as optimal for the breadmaking process because it leads to an improvement of the loaf volume, crust and crumb color, overall acceptability and also imparts a pleasant nutty flavor [150,151,152,153]. On the other hand, high proportions of the addition, above 20%, show a negative influence because a denser crumb structure, a reduced elasticity of the dough and a lower sensory acceptability are obtained. These things happen because of gluten dilution, concomitantly a decrease in the capacity of the dough to retain fermentation gases [151,152,153]. Taking these considerations into account, it can be concluded that the use of amaranth flour requires some additional actions to adjust technological parameters during breadmaking, such as more intense mixing and a longer dough proofing time, and it must be taken into account that the high fiber content leads to an increase in dough water absorption [152,154]. Taking into account the nutritional aspect, bakery products obtained with the addition of amaranth flour recorded an improvement in this regard, obtaining a greater amount of lysine, minerals such as calcium, magnesium and iron, proteins, ash and lipids, and a lower amount of carbohydrates [150,152]. The size of the flour particles also influences the characteristics of the bread. It is recommended to use medium-sized particles (180–300 μm) [151]. Also, this type of flour has been incorporated into other bakery products. For example, chapatti prepared with whole wheat and amaranth flour (20–50%) recorded an improvement in in vitro protein digestibility. Also, higher levels of lysine, fat, protein and minerals, and good sensory and textural properties were recorded. For this, it is recommended to use an addition of 40% amaranth flour [155]. In the case of cakes, the optimal addition level is 25%, which resulted in an improvement in color, texture and overall sensory characteristics [156].

It is observed that amaranth flour is also used in combination with other types of gluten-free flour to obtain gluten-free bakery products with improved nutritional quality. For example, the incorporation of amaranth flour in rice-based gluten-free bread resulted in an improvement in specific volume and porosity and a reduction in firmness. Furthermore, the partial replacement of rice flour and starch with up to 20% amaranth flour resulted in an increase in protein and mineral content. It was also found to slightly affect dough rheology and crumb texture [157,158]. Muffins prepared with black rice–amaranth flour mixtures showed increased levels of crude fiber, protein, fat and antioxidant activity as the proportion of amaranth used increased [159]. Calderón de la Barca et al. (2022) showed that a formulation containing 68.7% wheat flour, 22.7% amaranth flour and 8.6% sweet potato allows the production of bread with optimal textural, sensory and nutritional qualities [160].

### 5.3. Quinoa

Quinoa flour is often used in bakery formulations as an addition in different proportions, depending on the type of product, including up to 40% in bread and noodles, 60% in cakes and up to 70% in biscuits [23,161,162]. Referring to the technological aspect, the incorporation of this type of flour in wheat-based bread formulations has an influence on both the dough and the bread. A level of approximately 20% quinoa flour addition is considered optimal, taking into account the textural and sensory point of view. The addition results in an increase in protein, lipid, ash and total polyphenol contents and a reduction in starch content and starch digestibility due to the presence of dietary fiber and polyphenolic compounds [163,164]. Higher percentages of addition result in a reduction in loaf volume and an increase in crumb density due to the absence of gluten and weaker dough structure [165,166,167]. Of course, quinoa flour also influences dough rheology. Although it does not contain gluten-forming proteins, dough with quinoa flour had relatively stable pasting properties during heating and mixing [165,166]. The technological behavior of bakery products is influenced by the smaller starch granule size and lower amylose content of quinoa starch, which led to increased swelling power and water absorption capacity [166].

Quinoa flour in fermented form or quinoa sourdough has been reported to improve the quality of bakery products. The fermentation process improves the bread volume and delays staling through the formation of organic acids that inhibit starch retrogradation [168,169,170]. The fermentation process also has a desirable effect on the nutritional profile because it enhances the bioavailability of nutrients such as vitamins, minerals, amino acids and polyphenols and also reduces the amount of antinutritional compounds such as lectins, phytic acid, tannins and protease inhibitors. During fermentation, organic acids inhibit starch retrogradation, decreasing the staling rate from quinoa bread. Moreover, organic acids can synthesize dextran which behaves as a hydrocolloid to improve dough capacity to retain water and increase bread porosity [169,170]. The addition of quinoa flour has been used in many products, such as biscuits, where a percentage of 20–30% quinoa has been shown to result in a softer texture [22]. In gluten-free bakery products, quinoa flour is most often found in combination with rice flour, corn starch or other protein sources. These products, obtained with the addition of enzymes such as protease and lipase or with the addition of starch and dairy proteins, have been noted for their improved quality. Smaller starch granules and lower amylose content of quinoa starch are more susceptibility to enzyme activity which may have a positive impact and increase volume in gluten-free baked products [171,172,173]. One study showed that the addition of 10% quinoa flour in gluten-free bakery products based on rice and oat flour improved nutritional value without negatively affecting sensory acceptability [174]. Hussein et al. reported the production of gluten-free pan bread using quinoa flour and *spirulina* algae powder. The results of the study showed that technological strategies need to be adopted in such a way that the quality of the bakery products is not negatively affected [175].

### 5.4. Formulation Strategies and Their Impact on Bakery Products Quality

Studies show that there has been a recent trend in the use of fermentation in baking because sourdough fermentation helps to obtain products with improved sensory properties. In a study by Jagelaviciute and Cizeikiene (2021) [176], which highlighted the influence of non-traditional sourdough made with quinoa, hemp and chia flour on the characteristics of gluten-free maize/rice bread, it was concluded that the fermentation process can be successfully used to improve the porosity of bread samples, reduce the rate of bread staling and crumb firmness, and increase the overall acceptability of the bread. The decrease in crumb firmness can be explained in terms of a high water binding capacity. The positive effect of the fermentation process is mainly based on the following considerations: lactic acid bacteria produce free amino acids, flavor precursors, change the fiber solubility and degrade phytates and starch. Also, the resulting antimicrobial substances and organic acids lead to an increase in the shelf life of the bread. At the same time, the bacterial strains can produce exopolysaccharides such as dextran, reuteran and fructan, which lead to an increase in the viscosity of the dough and have the effect of decreasing the hardness of the crumb, increasing the porosity and slowing down the staling process of the bread [176,177]. The production of gluten-free bakery products has always posed a challenge for researchers due to the lack of the specific viscoelastic properties of gluten, which results in doughs with low cohesiveness and finished products with reduced volume, a crumbly texture, and poor color. To address these shortcomings, research in the field has focused on the use of gluten-free flours in combination with hydrocolloids, emulsifiers, and various types of enzymes. For example, in a study conducted by Mohammadi et al. (2014) [178], which analyzed the possibility of producing gluten-free flat bread using hydrocolloids, it was highlighted that xanthan gum and carboxymethyl cellulose significantly decreased the firmness of both fresh and stored breads. The maintenance of the softness of the bread and the extension of the shelf life are closely related to the water retention capacity which leads to an increase in moisture content and has the effect of delaying the process of starch retrogradation and bread hardening. Hydrocolloids have an important role in this process because they modify the structure of the starch, with an effect on the increase in water retention, which reduces the stiffness of the crumb. The addition of hydrocolloids also improved the elasticity of the crumb due to the capability of hydrocolloids to maintain water and due to the interactions with water. The fact that the addition of hydrocolloids resulted in the obtaining of bread with a lighter color for crumb and crust can be explained in terms of the water distribution that had an effect on Maillard browning and caramelization. The improvement of the porosity of gluten-free bread is explained by the fact that hydrocolloids contain hydrophilic and hydrophobic groups that are distinguished by their interfacial activity during dough fermentation and the formation of gel networks during fermentation and the baking process. In general, studies indicate that hydrocolloids improve the texture, increase the moisture content and extend the overall quality of gluten-free bread [178,179]. Also, to reduce the shortcomings brought about by the lack of gluten in the case of gluten-free bread, studies in the field have highlighted that a good solution is the use of enzymes. For example, in a study that analyzed the obtaining of gluten-free breads from cormels flour, it was concluded that the protease enzyme improved the specific volume of the bread. This is based on the fact that the enzyme presents the ability to modify protein functionality. The addition of protease led to the hydrolysis of proteins, resulting in smaller and more hydrophilic molecules, which contributed to better water retention in the sample. Protein hydrolysis creates smaller peptides and amino acids, which retain more water. This additional water hydrates the dough better, makes it more elastic and allows the formation of a more aerated structure in the bread. As a result, the specific volume of the bread increases compared to samples without the addition of protease [180]. The incorporation of quinoa into bakery products can meet consumer demands for functional, high-quality, and nutritionally enriched foods, even for gluten-free formulations. Various examples of bakery products obtained with pseudocereal flour are shown in Table 2.

## 6. Conclusions

Pseudocereals such as buckwheat, amaranth, and quinoa present a high nutritional value, being of high interest for their incorporation into bakery products. They are gluten-free grains with high-quality proteins, dietary fibers, essential minerals, and bioactive compounds such as phenolic compounds, saponins, phytoecdysteroids, phytosterols, betalains, peptides and bioactive proteins. Their use in bakery products enhances their nutritional profile by increasing both the quantity and quality of the nutrient compounds in the final products, contributing to potential health benefits against cardiovascular diseases, diabetes, cancer, high blood pressure, etc. Their functional properties make them suitable for bakery product applications with good-quality characteristics from the technological, nutritional and sensory point of view. However, their incorporation is often limited to a certain amount in bakery products due to their gluten-free nature, which affects dough rheological behavior and baked goods quality. Optimal substitution levels vary by bakery product type and pseudocereal. Generally, buckwheat is recommended to be incorporated at 10–20% in wheat-based breads and up to 85% in gluten-free blends, amaranth at 10–30% with sensory preference often around 20%, and quinoa at 5–20% in wheat breads, with higher-level addition in gluten-free breads and when technological adjustments are applied. Because pseudocereals are gluten-free, this makes them suitable for people suffering from celiac disease or gluten sensitivity, further increasing market demand. Their combination with other gluten-free raw materials allows food producers to obtain bakery products without gluten. Gluten-free products generally require hydrocolloids or enzymes to compensate for the absence of gluten, ensuring acceptable bakery product quality. Although today there are numerous studies that focus on a detailed analysis of the nutritional and functional properties of pseudocereals such as quinoa, amaranth, and buckwheat, several important knowledge gaps remain. Most studies in the field either only address the nutritional part in relation to health benefits or only isolated technological applications, and no comparative analysis is carried out for chemical composition, bioactive profile, technological functionality and measurable health outcomes in bakery products under the same working conditions in order to obtain the food product. It is also noted that in the specialized literature there is an important heterogeneity in terms of differences in substitution levels, flour particle size, processing conditions (such as fermentation type, germination, dough mixing) and analytical methodologies. This heterogeneity limits the comparability of results and prevents the formulation of standardized recommendations for the optimal incorporation of pseudocereals into bakery systems. A deeper mechanistic study of protein–starch–fiber interactions should also be performed under the same working conditions, on each pseudocereal in part, without using other additives such as enzymes or hydrocolloids in the dough matrix. There is also a limited existence of well-designed clinical studies to evaluate the long-term health effects of consuming pseudocereal-enriched bakery products. Studies are usually conducted in vitro or on animals, the analysis is rarely done with reference to humans, and studies are not conducted over a longer period of time.

## Figures and Tables

**Table 1:** Chemical composition of buckwheat, amaranth and quinoa.

Chemical Compounds	Buckwheat	Ref.	Amaranth	Ref.	Quinoa	Ref.	Wheat	Ref.
Protein (g/100 g)	5.7–16.4	[81]	12–22	[82,83]	13.1–14.1	[81,84]	10–14	[85]
Lipids (g/100 g)	3.4–7.4		6–13		4.7–5.5		2–3	
Carbohydrates (g/100 g)	67.8–81.4		65–75		53.7–57.2		70–75	
Fibers (g/100 g)	10.0–21.5		9–14		7–14.7		10–13	
Amino acids (g/100 g protein)								
Lysine	5.9–6.1	[81]	5.7	[86]	0.0445	[87]	0.335	[1]
Methionine	2.5–3.7		3.3		0.0079		0.201	
Tryptophan	1.4–2.0		1.9		-		0.16	
Leucine	6.4–6.7		6.2		0.0559		0.854	
Vitamins (mg/100 g)								
Thiamin (Vit.B1) (mg/100 g)	0.4–3.3	[81]	0.03–0.09	[88,89,90,91,92]	0.5–0.8	[81]	0.383	[1]
Riboflavin (Vit.B2) (mg/100 g)	0.2–10.6		0.01–0.07		0.2–0.3		0.115	
Niacin (Vit.B3) (mg/100 g)	6.2–18.0		0.1042		1.6–1.7		5.46	
Pantothenic acid (Vit.B5)	0.4		1.46	[93,94]	0.6		0.954	
Pyridoxine (Vit.B6)	0.6		0.59		0.2		0.6	
Folate (Vit.B9)	0.054–0.1		0.082		0.001–0.054		0.038	
α-Tocopherol (Vit.E)	0.3		1.19		2.1		1.01	
Minerals (mg/100 g)								
Calcium (Ca)	110	[81]	146.3–200	[88,89,90,91,92]	54	[81]	29	[1]
Iron (Fe)	2.2–4		6.53–66		5.3–7.5		3.19	
Magnesium (Mg)	231.0–390.0		246.62–328		119.0–227.0		41	
Phosphorus (P)	330.0–347.0		473.125–663		212.0–527.0		129	
Manganese (Mn)	1.3–3.4		0.88–5.71		1.8		1.03	
Zinc (Zn)	0.8–2.4		2.89–11.3		3.3–3.6		1.04	
Potassium (K)	450.0–460.0		400.5–552		474.0–649.0		141	
Copper (Cu)	0.5–1.1		0.28–1.07		0.2–0.5		0.148	

**Table 2:** Published studies on bakery products which different pseudocereals in their recipe.

Pseudocereals	Food Product	Main Raw Materials	Study Conclusions	Ref.
Buckwheat	wheat bread	wheat flour partially replaced with 5–30% buckwheat flour	higher antioxidant activity, phenolic content, protein, mineral content; slight loaf volume decrease for samples with higher amounts of buckwheat flour addition; acceptable sensory quality up to 20% substitution	[132]
		wheat flour partially replaced with 10–50% buckwheat flour	higher protein content, antioxidant activity, inositol phosphates, amino acid score (lysine limiting); texture and bread crumb characteristics improved at moderate substitution level (10–30%)	[51]
	wheat bread with different particles sizes of buckwheat flour	wheat flour, optimum level of buckwheat flour at different particle sizes (large ~9.13%, medium ~10.57%, small ~10.25%)	improved bread volume, porosity, elasticity; higher protein, lipids, amino acids, minerals content; best sensory scores for medium and large buckwheat flour particle sizes	[134]
	wheat bread with buckwheat sourdough	wheat flour, buckwheat sourdough (10–20%)	higher specific volume, softer crumb at 10% buckwheat sourdough addition; higher polyphenols content; extended shelf life	[181]
	wheat buns	wheat flour, buckwheat flour or sprouted buckwheat flour (10–30%), guar gum	higher nutritional value (protein, fiber, phenols, antioxidants), good sensory scores (especially for buns with 20% buckwheat, 10% sprouts)	[135]
	bread, crackers	wheat flour, sprouted buckwheat (10–30%, sprouted 48 h/72 h)	bread with improved volume, softer crumb; crackers comparable to control at up to 30% sprouted buckwheat addition	[137]
	cakes	wheat flour, buckwheat flour (5–20%)	higher protein, fiber, minerals content, antioxidant activity and comparable sensory scores to local branded cakes for samples with moderate buckwheat levels addition	[136]
	crackers	replacement of wheat flour with 20% buckwheat flour and buckwheat sourdough	higher phenolics, antioxidant activity, improved taste, chewiness and good sensory characteristics in especially with the use of buckwheat sourdough with starter culture *L. plantarum* or *L. brevis*	[182]
	premix for gluten-free breadmaking	buckwheat flour (88.2%), chia flour (9.8%), xanthan gum (2%)	increased the volume of the bread samples, higher hardness; higher protein, crude fiber content, antioxidant activity, essential polyunsaturated fatty acids (linoleic and linolenic acids)	[142]
	gluten-free bread	chia seed flour (10%), tartar buckwheat flour (90%)	bread with improved functional properties	[143]
		buckwheat flour, rice flour, potato starch (varied mixes via mixture design)	mixes with rice flour improved loaf volume, crumb softness, and sensory acceptability; even high amount of buckwheat (85%) lead to samples of a good quality	[175]
		rice, tapioca, 90:10 buckwheat:chia mix	higher flavonoid content, antioxidant activity, omega-3 fatty acids, acceptable volume, texture, good sensory scores	[143]
		mixed rice:buckwheat flour of 90:10, 80:20, 70:30 (husked and unhusked buckwheat)	rheological behavior similar to wheat dough, water absorption higher with unhusked buckwheat, higher hardness values for samples with high amounts of buckwheat flour, sensory scores acceptable for all samples	[183]
		mixed rice:buckwheat flour levels (10–30%)	higher specific volume, improved crumb cell structure, sensory acceptability for all bread samples	[144]
	gluten-free biscuits	buckwheat flour, sugar, hydrogenated vegetable fat, different types of gums (guar, acacia, xanthan and tragacanth) 1 g/100 g to buckwheat flour	higher oil absorption capacity (1.80 g/g) compared to the wheat flour biscuit samples (1.69 g/g), improved water absorption capacity and the emulsifying capacity of the flour through gums addition, good color, appearance, flavor and overall acceptability for biscuits with xanthan gum addition; good sensory scores	[146]
	gluten-free cookies	rice: buckwheat flour (10–30%)	higher antioxidant capacity, mineral content, sensory acceptability; germinated buckwheat delayed starch retrogradation	[147]
	gluten-free cakes (Seolgitteok)	rice, germinated buckwheat/pseudocereal flour	higher antioxidant activity, improved texture, and acceptable sensory properties	[103]
Amaranth	wheat bread	wheat flour (80%), amaranth flour (20%)	specific volume was reduced from 3.91 to 3.78 g/cm^3^, and the sensory characteristics (aroma, appearance, taste, texture, overall acceptability) decreased; the overall acceptability decreased from 8.00 to 4.22	[184]
		wheat flour, amaranth flour (10–30%)	specific volume and bread physical characteristics decreased; the number of pores/cm^2^ increased	[185]
	wheat bread with different particles sizes of amaranth flour	white wheat flour, amaranth flour (10–13%) with large particles, AL (>300 µm, <500 µm), medium, AM (>180 µm, <300 µm) and small, AS (<180 µm)	finer amaranth flour improved dough stability; coarse flour increased water absorption but decreased loaf volume; crumb porosity and sensory scores correlated with particle size; the bread obtained from composite flour with medium particle size was the most appreciated in terms of appearance, taste, smell, overall acceptability level; the volume of bread obtained from composite flour with average particle size was similar to that of the control bread; the bread obtained from composite flour with large and small particles showed lower values; nutritional content increased (protein, minerals); dough with higher amaranth showed decreased elasticity; bread was enhanced in protein, fiber, and mineral content; moderate substitution improved sensory acceptability	[151,152,153]
	chapatti (flatbread)	wheat flour, amaranth flour (10–15%)	amaranth increased protein and fiber content; dough required slightly more water, chapatti hardness slightly increased, but sensory quality remained high	[155]
	cookies	wheat flour, amaranth flour (10–20%)	cookies with 10–15% amaranth were sensory acceptable; higher substitution increased hardness; protein and mineral content improved; slight reduction in spread ratio	[156]
	gluten-free bread	rice flour, tapioca starch, amaranth, buckwheat, quinoa, xanthan gum (0.3% of flour basis), carboxymethylcellulose (0.3% of flour basis)	optimized multi-pseudocereal blends improved specific volume, loaf height, and crumb structure; nutrient composition had increased (proteins, fiber, minerals, antioxidants) and overall acceptability was high	[133]
	gluten-free bread (with enzymes)	rice flour, corn starch, potato starch or tapioca starch, hydrocolloids: xanthan gum or guar gum, amaranth flour (10–30%), enzymes (lipase, protease)	amaranth flour improved nutrient value of bread (protein, minerals, antioxidants); enzymes improved dough handling, crumb structure, and loaf volume; sensory attributes were acceptable across substitutions	[157]
	gluten-free flat bread	rice flour, corn starch, tapioca or potato starch, xanthan gum, amaranth flour (10–30%)	enriched flat bread had higher protein, fiber, and minerals content; moderate substitution (20%) presented best sensory score; higher substitution increased hardness	[158]
	gluten-free muffins	black rice, corn starch or potato starch, amaranth flour (10–25%)	higher protein, fiber, and minerals content; texture improved (softer crumb, less staling); acceptable sensory profile even at higher substitution level	[159]
Quinoa	wheat bread	wheat flour, quinoa flour (10–40%)	the best bread quality was for the 20% quinoa flour incorporated in bread recipe; higher amounts of quinoa addition decrease loaf volume and increase firmness; higher protein and fiber content, decrease in dough elasticity and in vitro starch digestibility, which decreases glycemic index	[42]
		wheat flour, quinoa flour (10%)	bread sample presented similar quality to white bread; pasting properties of the flours are more stable during heating and mixing, the disintegration of the gelatinized starch granule was less when quinoa was added in dough recipe	[165]
		wheat flour, quinoa flour (10, 20%)	slightly lower volume than control bread, denser texture, higher nutritional value, acceptable sensory characteristics	[164]
		wheat flour, quinoa flour (5–15%)	5% quinoa flour did not negatively change the bread sample; 10–15% negatively influenced the quality of the bread, decreasing the specific volume and hardness, but was positively appreciated for its taste	[167]
		wheat flour, quinoa flour (0–20%)	protein, fiber, ash, and fat increased with quinoa; water absorption and swelling increased; loaf volume decreased gradually but bread up to 10% quinoa was acceptable in sensory quality; 15–20% reduced acceptability despite higher nutrition	[161]
		wheat flour, quinoa flour (5–20%)	quinoa addition increased protein, fiber, and minerals; higher firmness and lower loaf volume with higher quinoa flour; 5–15% quinoa flour led to acceptable baked texture and improved nutritional value	[167]
	cookie, bread, steamed bread quality	wheat flour, quinoa flour (0–90%)	increasing quinoa reduced gluten networking (weaker structure); optimal around 20% quinoa for dough rheology; increasing the amount of quinoa flour in bread increased darkness and redness of the samples and decreased yellowness; hardness increased, whereas chewiness and cohesiveness decreased, less mold growth by quinoa flour addition	[186]
	wheat bread with quinoa sourdough	wheat flour, fermented using *L. brevis* (5–10%), unfermented quinoa (5%) 5% unfermented quinoa	bread porosity decreased from 78.1% to 77.7% for the bread sample with 10% quinoa addition, and specific volume decreased from 3.09 to 3.32 cm^3^/g; from a sensory point of view, the samples with 5% fermented quinoa flour were the most appreciated in terms of overall acceptability, compared to the addition of non-fermented quinoa flour	[169]
	crispy biscuits	wheat flour, quinoa flour (10–30%)	biscuits obtained presented a low hardness and firmness, having a softer texture, an aspect that may negatively influence the perception of consumers	[22]
	gluten-free bread	gluten-free flour mix (rice flour/corn starch, 50:50), guar gum, fermented buckwheat, quinoa, amaranth (0–45%)	higher protein, ash, total phenolic content, antioxidant activity, mineral content Ca, P, K, Fe, Mg, Zn by 1.3–4.3 times; softer crumb with the best acceptable quality up to 30% fermented pseudocereals flours	[168]
		quinoa flour, starch, dairy protein (sodium caseinate, whey protein isolate), different mixes	addition of starch and dairy proteins improved texture and quality parameters of quinoa-based gluten-free bread; higher specific volume, crumb firmness when quinoa flour was incorporated with potato or corn starch and also when sodium caseinate was associated with corn starch	[173]
	gluten-free bread with enzymes	rice flour, corn starch, sodium caseinate, inulin, DATEM, xanthan gum, quinoa flour (0–25%), enzymes (protease, lipase)	enzymes (e.g., lipase/protease) improved gluten-free bread quality (volume, texture) with 15% quinoa substitution in bread; samples with substitution of quinoa had a darker crust color; using 15% quinoa in bread recipe did not change the porosity percentage	[171]
	gluten-free cake batters, cake quality	quinoa flour/rice flour/potato starch (0/50/50, 25/37.5/37.5, 50/25/25, 75/12.5/12.5)	quinoa improved batter stability, mechanical strength, homogeneity, sensory and nutritional characteristics of the products; up to 50% quinoa flour addition can be incorporated into gluten-free cake formulations without negatively affecting it quality	[187]

## Data Availability

No new data were created or analyzed in this study. Data sharing is not applicable to this article.
